# The effect of egg supplementation on growth parameters in children participating in a school feeding program in rural Uganda: a pilot study

**DOI:** 10.1080/16546628.2017.1330097

**Published:** 2017-06-06

**Authors:** Jamie I. Baum, Jefferson D. Miller, Brianna L. Gaines

**Affiliations:** ^a^ Department of Food Science, University of Arkansas, Fayetteville, AR, USA; ^b^ Department of Agricultural Education, Communications and Technology, University of Arkansas, Fayetteville, AR, USA

**Keywords:** Children, Uganda, eggs, growth, mid-upper arm circumference, school feeding program, human nutrition

## Abstract

**Background**: School feeding programs have gained popularity in developing countries . Eggs are an inexpensive source of micronutrients and high-quality protein. Therefore, the objective of this study was to gain preliminary data regarding the impact of egg supplementation on growth in primary school students participating in a school feeding program in rural Uganda.

**Methods**: Children (ages 6–9; n = 241) were recruited from three different schools located throughout the Kitgum District of Uganda. All participants in the same school received the same dietary intervention: control (no eggs (0 eggs); n = 56), one egg five days per week (1 egg; n = 89), or two eggs five days per week (2 eggs; n = 96). Height, weight, tricep skinfold thickness (TSF), and mid-upper arm circumference (MUAC) were measured monthly over 6 months.

**Results**: Following six months of egg supplementation, participants receiving 2 eggs had a greater increase in height and weight compared to the 0 eggs and 1 egg groups (P < 0.05). In addition, participants receiving 1 egg and 2 eggs had a significantly higher (P < 0.05) increase in MUAC at six months compared to 0 eggs.

**Conclusion**: These results suggest that supplementation with eggs can improve parameters of growth in school-aged children participating in school feeding programs in rural Uganda.

**Abbreviations:** MUAC: Mid-Upper Arm Circumference; TSF: Tricep Skinfold Thickness

## Background

Although protein-energy malnutrition is a concern for many children in developing countries, micronutrient malnutrition has been recognized as a more widespread problem [1]. This prevalence of malnutrition is linked to poverty, poor diet quality, and little or no intake of animal-source foods [2,3]. Countries in sub-Saharan Africa have a high prevalence of children that are underweight, wasting, and stunting [4,5]. In Uganda, the prevalence of malnutrition remains highest among children younger than five years of age [4,5] and the nutrition status of children over the age of five in Uganda remains relatively unknown.

School feeding programs are gaining popularity in developing countries such as Uganda [6]. Such programs aim to reduce the short-term hunger that negatively impacts concentration span and learning capacity of school children [6]. A recent study found that early stage primary school children attending a Malawian school feeding program for one year had improved catch-up growth in lean muscle mass and improved cognitive outcomes compared to children attending a non-school feeding program school [7].

Eggs are an inexpensive source of 13 essential micronutrients (i.e. choline, biotin, riboflavin, vitamin B12, pantothenic acid, vitamin A, folate, vitamin E, vitamin D, calcium, magnesium, sodium, phosphorus, potassium, and zinc), and an excellent source of high-quality protein [8,9], which makes them an ideal candidate for implementation into a school feeding program. Therefore, the objective of this study was to gain preliminary data regarding the impact of egg supplementation five days per week on growth in primary school students participating in a school feeding program in rural Uganda. Growth outcomes are a relatively easy, non-invasive measure to evaluate the effects of eggs on nutrition status, which would support future studies on the effects of eggs on cognitive performance. We hypothesized that egg supplementation with eggs will improve markers of growth.

## Participants and methods

### Participants

Participants were recruited from three different schools located throughout the Kitgum District of Uganda (Amide, Palabek, and Padibe). Participants were recruited with the help of the director over the three schools. The participants were required to be between six and eight years of age. There were no health restrictions for receiving eggs. Every student in each class was provided eggs in order for children not to feel excluded from their peers. However, participants who missed data collection points, did not eat eggs, or were known to be HIV-positive were excluded from the final data analysis. Data were collected at baseline (one to two weeks before the start of intervention), and each month for the six-month duration of the study. The data collection timeline was the same for each school. Ethical approval for the study was obtained from the Institutional Review Board at the University of Arkansas (Fayetteville, AR; #13-12-377). Written or verbal consent was obtained from legal guardians of all participants and written or verbal assent was obtained from all children who participated in the study.

### Dietary intervention

All participants within the same school received the same dietary intervention: control (no egg supplementation (0 eggs)), one egg supplemented five days per week (1 egg), or two eggs supplemented fives days per week (2 eggs). Eggs were served hardboiled and consumed as a snack during the school day. Eggs were provided by the One Egg non-profit organization (Cordova, TN) and were delivered to schools on a weekly basis.

### Anthropometric measurements

Weight, height, tricep skinfold thickness and mid-upper-arm circumference were measured monthly. Body weight was measured to the nearest 0.5 kg with participants barefoot using a standard calibrated digital scale. Height was measured to the nearest 0.1 cm using a standing stadiometer with participants barefoot, in the free-standing position. Tricep skinfold thickness and mid-upper arm circumference were measured according to the protocols outlined by the NHANES Anthropometry Procedures Manual [10]. All measurements were taken in duplicate. Nurses present at each school were trained by the investigators and provided with an instruction manual to take the anthropometric measurements to ensure consistency.

### Statistical analysis

All data were analyzed using one-way ANOVA within each time point. Two-way ANOVA was used to measure changes between groups over time. Results are reported as means ± SEM. All analyses were conducted using Prism GraphPad Software Version 6.0 (La Jolla, CA). *P *< 0.05 was considered statistically significant. There was no difference in age, weight, and height between males and females within intervention groups, therefore male and female data were combined to analyze changes from baseline.

## Results

### Participant characteristics

**Figure 1.**
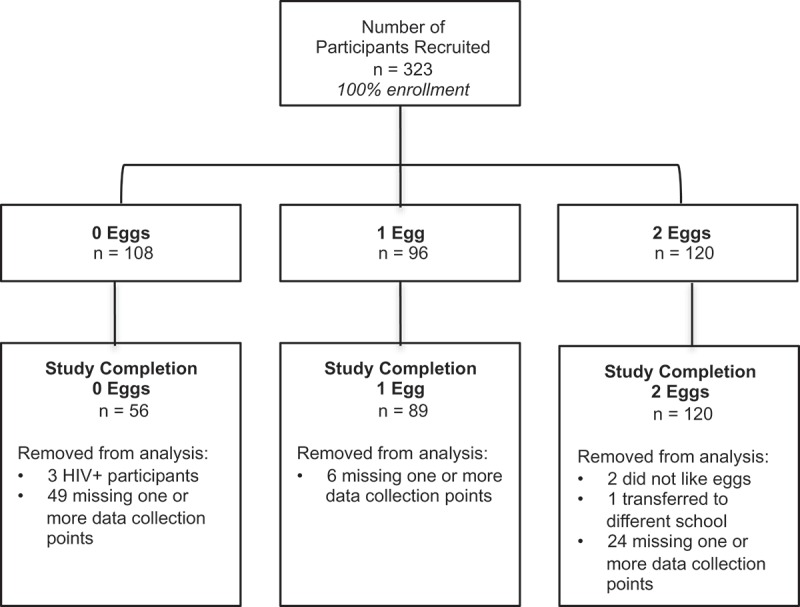
Participant enrollment and retention flow diagram.

Baseline participant characteristics by sex are presented in Table 1. There was no difference in baseline age, weight, and height between males and females within intervention groups. There were 323 participants recruited for the study and 241 completed all data collection points (Figure 1). The number of participants completing the intervention were as follows: 0 egg group, n = 53; 1 egg group, n = 89; and 2 eggs group, n = 96. Three participants were excluded from the 0 egg group data analysis because they were known to be HIV positive and 52 participants had incomplete data (e.g. missing one or more data collection points). In the 1 egg group, 6 participants were excluded from data analysis due incomplete data, and in the 2 eggs group 24 participants were not included in data analysis due to incomplete data, two did not eat eggs and one participant transferred to a new school during the intervention. There was a significant difference in starting age between the 0 eggs (7.2 ± 0.1 y) and 2 eggs (6.5 ± 0.1 y) groups (*P *< 0.001) and the 1 egg (6.9 ± 0.1 y) and 2 eggs groups (*P *< 0.01). There was a significant difference in baseline weights between the 0 eggs (19.4 ± 0.3 kg) and 1 egg (21.5 ± 0.8 kg) groups (*P *< 0.05) and the 0 eggs and 2 eggs (18.9 ± 0.3) groups (*P* < 0.001). Finally, there was significant difference in baseline height between the 0 eggs (119.2 ± 1.0 cm) and 2 eggs (115.1 ± 0.6 cm) groups (*P* < 0.001) and the 1 egg (117.7 ± 0.7 cm) and 2 eggs groups (*P* < 0.05).

**Table 1.** Participant characteristics^1.^

	0 eggs		1 egg		2 eggs	
	n = 56		n = 89		n = 96	
	Male	Female	Male	Female	Male	Female
Sample size, n	32	24	40	49	44	52
Age, y	7.0 ± 0.2^ac^	7.4 ± 0.2^a^	7.0 ± 0.1^ac^	6.8 ± 0.1^ac^	6.4 ± 0.1^bc^	6.6 ± 0.1^c^
Height, cm	117.5 ± 1.1^ab^	121.2 ± 1.6^a^	118.2 ± 1.1^ab^	117.3 ± 0.8^ab^	114.9 ± 0.9^b^	115.3 ± 0.8^b^
Weight, kg	20.0 ± 0.4^ab^	21.5 ± 0.8^a^	20.0 ± 0.5^b^	18.9 ± 0.4^abc^	19.0 ± 0.4^c^	18.8 ± 0.4^bc^

^1^Data expressed as means ± SEM. Values not sharing the same letter are significantly different within a row (*P *< 0.05).

### Anthropometric measurements

**Figure 2.**
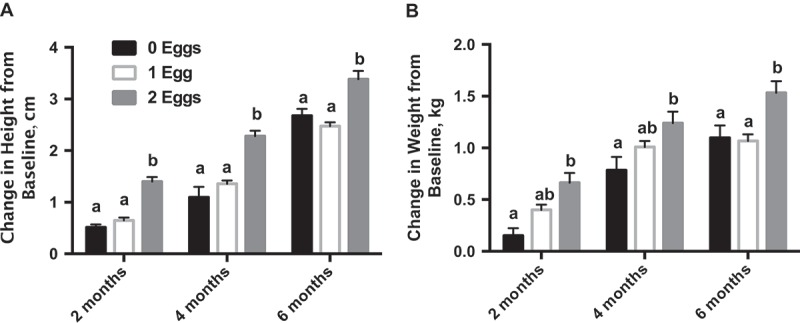
The effect of egg supplementation on height and weight over six months. (a) Change in height from baseline at two, four4 and six months. (b) Change in weight from baseline at two, four, and six  months. Data expressed as means ± SEM. Values not sharing the same letter are significantly different within a time point (*P *< 0.05).

Although all participants increased in height and weight over the duration of the study, participants in the 2 eggs group had significantly higher growth (*P *< 0.05) and weight gain (*P* < 0.05) at the end of the sixon e-month intervention compared to the 0 eggs and 1 egg groups (Figure 2). The results for height, weight, tricep skinfold (TSF) and mid-upper arm circumference (MUAC) are presented in Table 2. There was a significant difference in change in TSF between baseline and 6 months between 0 eggs (+0.67 ± 0.21 cm) and 1 egg (−0.73 ± 0.14 cm; *P* < 0.001) and 0 eggs and 2 eggs (−0.64 ± 0.11 cm; *P *< 0.001), but not between 1 egg and 2 eggs. There was a significant difference in change in MUAC between baseline and 6 months between 0 eggs (+0.18 ± 0.11 cm) and 1 egg (+0.63 ± 0.09 cm; *P* < 0.01) and 0 eggs and 2 eggs (+0.52 ± 0.07 cm; *P *< 0.05), but not between 1 egg and 2 eggs.

**Table 2.** Change in growth parameters over six months.^1^

	0 Eggs		1 Egg		2 Eggs	
	Male	Female	Male	Female	Male	Female
**Baseline**						
Height, cm	117.5 ± 1.1^ab^	121.2 ± 1.6^a^	118.2 ± 1.1^ab^	117.3 ± 0.8^ab^	114.9 ± 0.9^b^	115.3 ± 0.8^b^
Weight, kg	20.0 ± 0.4^ab^	21.5 ± 0.8^a^	20.0 ± 0.5^b^	18.9 ± 0.4^abc^	19.0 ± 0.4^c^	18.8 ± 0.4^bc^
TSF, cm	5.3 ± 0.2^a^	7.1 ± 0.4^b^	6.3 ± 0.3^ab^	7.1 ± 0.3^b^	5.0 ± 0.2^ac^	5.7 ± 0.2^a^
MUAC, cm	17.0 ± 0.1^ab^	17.6 ± 0.3^b^	16.6 ± 0.2^ac^	16.6 ± 0.2^ac^	15.9 ± 0.2^c^	15.9 ± 0.1^c^
**2 months**						
Height, cm	119.2 ± 1.1^ab^	122.1 ± 1.7^a^	118.7 ± 1.2^ab^	117.8 ± 0.8^ab^	116.1 ± 0.9^b^	116.6 ± 0.8^b^
Weight, kg	20.4 ± 0.4^ab^	21.8 ± 0.8^a^	20.5 ± 0.5^ab^	19.3 ± 0.3^b^	19.6 ± 0.4^b^	19.5 ± 0.4^b^
TSF, cm	5.9 ± 0.2^a^	7.3 ± 0.4^b^	5.8 ± 0.2^a^	6.4 ± 0.2^ab^	5.7 ± 0.2^a^	6.3 ± 0.2^ab^
MUAC, cm	17.2 ± 0.1^ab^	17.7 ± 0.3^b^	16.7 ± 0.1^ac^	16.6 ± 0.1^ac^	16.4 ± 0.2^c^	16.5 ± 0.1^c^
**4 months**						
Height, cm	118.7 ± 1.1^ab^	122.5 ± 1.7^a^	119.5 ± 1.1^ab^	118.6 ± 0.8^ab^	117.5 ± 0.9^b^	117.4 ± 0.8^b^
Weight, kg	20.9 ± 0.5^ab^	22.5 ± 0.8^a^	21.0 ± 0.5^ab^	19.9 ± 0.3^b^	20.0 ± 0.4^b^	40.2 ± 0.3^b^
TSF, cm	6.1 ± 0.3^ac^	7.3 ± 0.4^bc^	6.2 ± 0.2^ac^	6.8 ± 0.2^ab^	5.5 ± 0.1^c^	6.3 ± 0.2^a^
MUAC, cm	17.2 ± 0.2^ab^	17.7 ± 0.3^b^	17.0 ± 0.1^ab^	16.8 ± 0.1^a^	16.4 ± 0.2^c^	16.6 ± 0.2^ac^
**6 months**						
Height, cm	119.2 ± 1.1^ab^	124.1 ± 1.6^a^	120.7 ± 1.1^ab^	119.7 ± 0.8^ab^	118.3 ± 0.9^b^	118.7 ± 0.8^b^
Weight, kg	21.3 ± 0.5^ab^	22.7 ± 0.8^a^	21.0 ± 0.5^ab^	20.0 ± 0.3^b^	20.5 ± 0.4^b^	20.3 ± 0.3^b^
TSF, cm	6.3 ± 0.2^a^	7.6 ± 0.4^b^	5.6 ± 0.2^a^	6.3 ± 0.3^a^	5.6 ± 0.1^a^	6.4 ± 0.2^a^
MUAC, cm	17.2 ± 0.2^abc^	17.9 ± 0.3^b^	17.3 ± 0.2^ab^	17.0 ± 0.3^abc^	16.3 ± 0.2^c^	16.5 ± 0.1^ac^

^1^Data expressed as means ± SEM. Values not sharing the same letter are significantly different within a row (*P* < 0.05). TSF: tricep skinfold thickness; MUAC: mid-upper arm circumference.

## Discussion

To our knowledge, this is the first study to examine the effects of egg supplementation in school-aged children in rural Uganda participating in a school feeding program. The results of this pilot study clearly support the concept that incorporating two eggs per day into school feeding programs can have a positive effect on markers of physical growth of children in rural Uganda.

Malnutrition in childhood has lasting effects on health and quality of life. Currently, 156 million children are stunted and another 50 million suffer from wasting [11]. In Africa, the number of stunted children is rising [11]. Therefore, it is important to develop effective nutritional strategies for prevention and treatment. One possible strategy is through meals containing high quality protein, such as animal protein. Very little data exists examining the effects of animal protein-source foods on outcomes of growth and development in school-aged children at risk for malnutrition in developing countries in sub-Saharan Africa, especially Uganda which has 17.5 million caloric insecure individuals in 2005/2006 [12]. This study found that supplementing young children living in rural Uganda with two-eggs per day, five days per week over six months resulted in a significant increase in height and weight compared to 0 eggs and 1 egg per day, which did not differ. These findings are supported by data from studies conducted in Kenya [3] and Guinea-Bissau [13], which found that supplementation with animal-source foods (e.g. meat and dairy) improved parameters of growth such as MUAC [3,13] and weight-for-age *z* score [13] in children. In the study conducted in Guinea-Bisseau, preschool children were provided ready-to-use supplementary foods (RUSF) with either 15%- or 33%-protein from dairy sources five days per week for three months [13]. Consumption of RUSFs with 33% protein content, versus 15% protein content, eliminated a decrease in MUAC when compared to children not receiving RUSFs. There was no difference in MUAC between 33%- and 15%-protein over the course of the study. In the current study, we observed a significant increase in MUAC following six months of supplementation with both one and two eggs. However, only supplementation with 2 eggs resulted in a significant increase in weight and height compared to 0 eggs and 1 egg. In another study, children from the rural Embu District of Kenya received githeri supplemented with either meat, milk or fat as a midmorning snack at school for nearly two years [3]. The children receiving meat had a doubling of upper midarm muscle area, while the milk group had a more modest increase compared to the control group (githeri with fat) [3,14]. The results observed above are similar to our data, which suggests that sources of high-quality protein, such as eggs, can promote large changes in growth indices in children at risk for malnutrition. Children in rural Kenya supplemented with either daily meat- or milk-based snacks at school had improved diet quality (e.g. micronutrient intake) and diet quantity (e.g. caloric intake) compared to children receiving no snacks or a portion of a vegetable stew (githeri) [1].

Since this was a pilot study, there were several limitations. The first limitation is that many participants did not have birth dates on record, so we used estimated age according to school records, therefore starting age, heights and weights varied from school to school, which may have influenced the results. Second, the school nurses were trained to collect data during months 2–6, which may have resulted in measurement errors in tricep skinfold thickness. In addition, the nurses were not blinded to the dietary interventions taking place at their schools. Due to the nature of this pilot study, we were not able collect food records so we could not assess overall nutrient intake. In addition, there was a three-week school break between months five and six in which the participants did not receive eggs, which may have impacted the final data collection points. Due to the rural location we were unable to collect blood samples so were not able to gain any insight into nutrient status. Finally, cognitive performance indicators were not measured so we are not able to make any conclusions related to school performance.

## Conclusions

The data from this study are important because there are several programs being launched in developing countries focuses on supplementation of one egg per day as a way to improve growth and development of young children. However, to date, no scientific data have been published. Despite the limitations to this pilot study, we were able to demonstrate that it is only with the supplementation of two eggs per day, five days per week that improves parameters of growth in school-aged children participating in school feeding programs in rural Uganda. However, further research is needed to determine the effects of egg supplementation on nutrient status and cognitive development, and to determine the feasibility of implementing egg supplementation into school feeding programs for an extended period of time. It is estimated that feeding 100 children one egg per day, fives days per week costs $5,000 US dollars [15], so we can assume that it will cost approximately twice that amount to feed children two eggs per day. Therefore, additional work is needed to determine the cost effectiveness of a long-term egg supplementation program in developing countries.
